# Microbial experience through housing in a farmyard-type environment alters intestinal barrier properties in mouse colons

**DOI:** 10.1038/s41598-023-40640-5

**Published:** 2023-08-22

**Authors:** Henriette Arnesen, Turhan Markussen, George Birchenough, Signe Birkeland, Elisabeth E. L. Nyström, Gunnar C. Hansson, Harald Carlsen, Preben Boysen

**Affiliations:** 1 Faculty of Veterinary Medicine, Norwegian University of Life Sciences (NMBU), Ås, Norway; 2 Faculty of Chemistry, Biotechnology and Food Science, Norwegian University of Life Sciences (NMBU), Ås, Norway; 3 Mucin Biology Group, Department of Medical Biochemistry and Cell Biology, University of Gothenburg, Gothenburg, Sweden

**Keywords:** Mucosal immunology, Biological models

## Abstract

To close the gap between ultra-hygienic research mouse models and the much more environmentally exposed conditions of humans, we have established a system where laboratory mice are raised under a full set of environmental factors present in a naturalistic, farmyard-type habitat—a process we have called feralization. In previous studies we have shown that feralized (Fer) mice were protected against colorectal cancer when compared to conventionally reared laboratory mice (Lab). However, the protective mechanisms remain to be elucidated. Disruption of the protective intestinal barrier is an acknowledged player in colorectal carcinogenesis, and in the current study we assessed colonic mucosal barrier properties in healthy, feralized C57BL/6JRj male mice. While we found no effect of feralization on mucus layer properties, higher expression of genes encoding the mucus components Fcgbp and Clca1 still suggested mucus enforcement due to feralization. Genes encoding other proteins known to be involved in bacterial defense (Itln1, Ang1, Retnlb) and inflammatory mechanisms (Zbp1, Gsdmc2) were also higher expressed in feralized mice, further suggesting that the Fer mice have an altered intestinal mucosal barrier. These findings demonstrate that microbial experience conferred by housing in a farmyard-type environment alters the intestinal barrier properties in mice possibly leading to a more robust protection against disease. Future studies to unravel regulatory roles of feralization on intestinal barrier should aim to conduct proteomic analyses and in *vivo* performance of the feralized mice intestinal barrier.

## Introduction

Throughout evolutionary history, mammals have co-evolved with the billions of microbes surrounding them and colonizing their bodies. The host and their microbiota have developed a symbiotic relationship fundamental for host fitness, emphasized by the major impact the microbiota have on host metabolism and development of organ systems, including the immune system^1–4^. Yet, laboratory mice used to model human responses are usually studied under strictly hygienic conditions, deprived of the natural stimuli a microbially rich environment provides. The dogma for laboratory mouse studies have long been to create a highly standardized environment with emphasis on genetic similarities and microbial control with strict surveillance of pathogen status. This has several advantages, but also creates a risk that laboratory mice are removed from their natural conditions, and also deviate from the organism they are aimed to model, humans, that rarely live under microbial isolation. Lately, an increased focus has been turned towards generating more naturalistic mice that can recapitulate realistic traits, aiming to improve the translatability from mouse models to human relevance^5^. We have established a model system where laboratory mice are feralized in a farmyard-type habitat, producing a real-life adapted mammal^6^.

In two different mouse models of colorectal cancer (CRC), we have shown that the feralized mice were protected against colorectal carcinogenesis when compared to conventionally reared laboratory mice^4^. This could potentially recapitulate benefits of a rural lifestyle among humans, such as that agricultural workers have lower incidence of several types of cancer, including colorectal cancer^7^. The intestinal barrier is the first line of defense and is thus very interesting with respect to colorectal carcinogenesis. The intestinal barrier encompasses the mucus layer and a single layer of epithelial cells tied together by anchoring structures to prevent paracellular passage of luminal content. Specialized cells such as mucus-producing Goblet cells, antimicrobial peptides-producing Paneth cells as well as intraepithelial lymphocytes are important components maintaining barrier integrity^8^. The organization of the mucus layer varies along the length of the intestines, reflecting the properties of the intestinal segments. The small intestine is lined with a single loosely organized layer allowing efficient nutrient uptake, but also leaving it penetrable by luminal microbes. The small intestine depends on antimicrobial molecules to keep bacteria away from the epithelium. The colon mucus layer is in contrast made up two layers; lined by an inner, dense layer and an outer loose layer, which physically prohibits close contact between bacteria and epithelium (inner layer) and provide a bacterial habitat (outer layer)^9^. The mucus layer is continuously renewing and is crucial to hinder luminal contents to contact the epithelial wall and underlying tissue as well as prevent bacterial overgrowth. Alterations in the intestinal barrier properties may lead to increased permeability facilitating encroachment of microbes and toxins to the epithelium, inflicting inflammation and tissue damage that in turn may promote CRC development^10^. Changes in the gut microbiome is increasingly recognized as playing a pivotal role in regulating intestinal barrier function, and the gut microbiota composition has been shown to shape the mucus layer in mouse colons^11^. Various strategies to introduce a more diverse and naturalistic microbial environment to laboratory mice have demonstrated significant changes in gut microbiotas and immune regulation^12–14^, and impact on systemic responses such as metabolic syndrome^15^. Moreover, fecal transfer of wild mouse microbiota has been shown to ameliorate CRC in laboratory mice^16^, highlighting the potential of an enhanced mucosal barrier conveying CRC protective effects in naturalized mice. Wild-caught mice have been shown to have a thicker and less penetrable mucus layer than conventional laboratory mice^17^. Yet, effects of naturalistic housing on mucus layer and the intestinal epithelium remains to be studied.

With the current study we aimed to address how mice introduced to a farmyard-type habitat (feralization) influenced intestinal mucosa in healthy mice by assessing the intestinal mucus layer properties and mucosal gene expression. To evaluate if potential effects of feralization on the mucosal barrier could be explained by differences in gut microbiota, we also characterized the caecal microbiota.

## Materials and methods

### Animals and housing conditions

A microbially enriched, semi-naturalistic model was designed at the Norwegian University of Life Sciences (NMBU) as previously described^4^. Briefly, to resemble the common habitat of the house mouse (*Mus musculus*), indoor pens were enriched with used livestock bedding, organic soil, straw and fecal content from ecologically farmed pigs, cows, horses and poultry.

**Figure 1:**
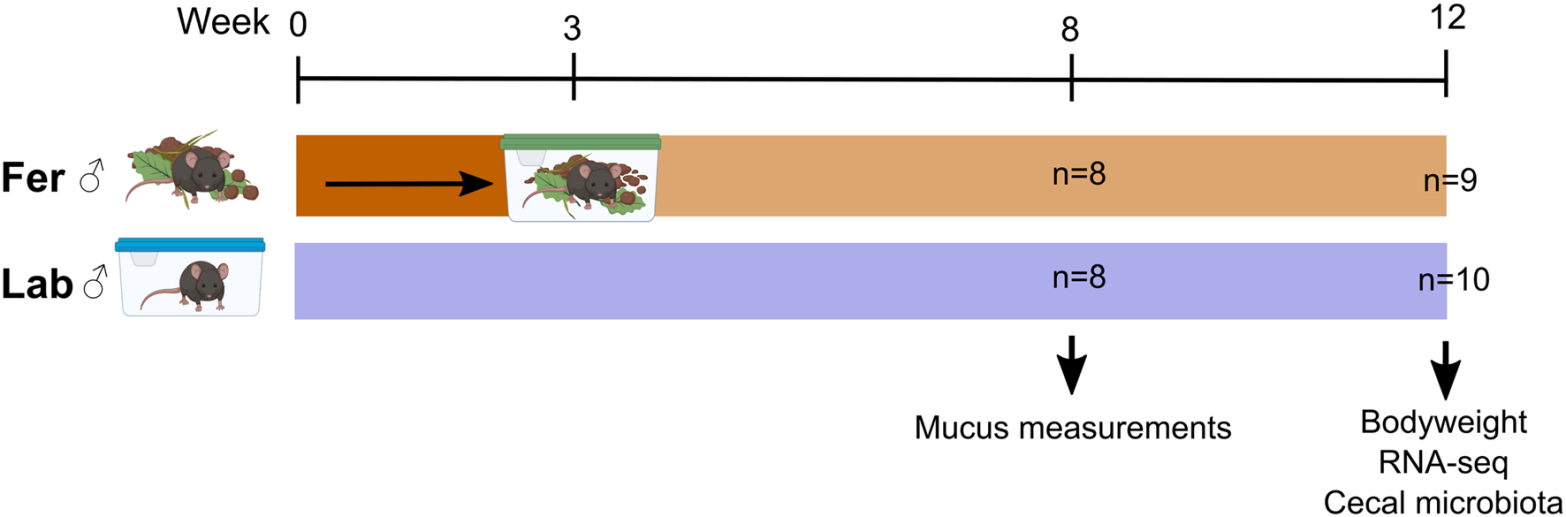
Experimental setup. 35 male mice were either born in a farmyard-type habitat (Fer, n = 17) or in conventional clean laboratory cages (Lab, n = 18). After weaning (week 3), the mice were randomized into two groups and moved to new cages. For the Fer mice this implied being moved from the farmyard-type habitat to cages containing the same microbially rich farmyard material.

The animal experiment was approved by the Norwegian Animal Research Authority (FOTS ID 18012) and is reported according to the ARRIVE guidelines (https://arriveguidelines.org). The study was conducted at NMBU and University of Gothenburg in accordance with local and national regulations for laboratory animal research. 30 female C57BL/6JRj (Janvier Labs, Saint-Berthevin Cedex, France) mice aged 3 weeks were feralized for 7 weeks prior to breeding. For breeding, two feralized females were brought together with one non-feralized, age matched C57BL/6JRj male (from the same supplier) in individually ventilated cages (IVCs; Innovive Inc., San Diego, CA, USA) enriched with feralizing pen material. After 10 days, the females returned to the pens to deliver. As controls, 24 female mice from the same batch were housed and mated under pathogen-free (“laboratory”) conditions. All mice were kept under standard 12 h light/dark cycle, 23–25 °C and 45–55% relative humidity conditions. Water and standard chow diet (RM1(E), SDS; Special Diet Services, Essex, United Kingdom) was provided ad libitum. At 3 weeks of age, male offspring (in total 35) were weaned and then housed under the conventional laboratory conditions (Lab, n = 18), or in cages enriched with environmental material from the mouse pens (feralized; Fer, n = 17) (Fig. 1). The female offspring were recruited to a separate experiment, where the influence of feralization on development of colorectal cancer was investigated^4^. The male Lab and Fer mice were sacrificed at 8 weeks of age for mucus measurements (n = 8 in both groups), or at 12 weeks of age for RNA sequencing and caecal microbiota profiling (Lab; n = 10, Fer; n = 9). Laboratory analyses were performed blinded towards group allocation.

### Mucus growth rate, thickness and penetrability

Colonic mucus growth rate was measured in an Ussing chamber-like ex vivo perfusion system using a micro needle and stereo microscope as previously described^18^. Mucus growth rate (representing secretion and proteolytic expansion) was expressed as µm mucus growth/minute. Baseline (pre-treatment; n = 8) growth rate was established for all samples before 50% (n = 4) were then treated with EDTA (metalloprotease inhibitor) and 50% (n = 4) were treated with 1X cOmplete EDTA-free protease inhibitor cocktail (serine + cysteine protease inhibitor mixture) (Roche) in the apical buffer for 30 min. The inhibitor treatment was conducted to assess potential differences between the groups in involvement of endogenous proteases in controlling mucus expansion^19^.

Colon and ileum mucus barrier properties were measured by assessing penetration of bacteria-sized beads via confocal microscopy according to previously established methodology^20^. Briefly, the tissue was stained, and microbeads were allowed to sediment onto the mucus for 5 min before the surface was gently washed to remove excess microbeads. The tissue and microbeads were visualized with microscopy using a LSM700 Axio Examiner Z.1 confocal microscope with Plan-Apochromat x 20/1.0 DIC water objective (Zeiss) and the Zen 2010 software (Zeiss). Barrier function was expressed as normalized penetrability (bead distribution within mucus) and mucus thickness as average tissue-bead distance. For ileum, mucus thickness was measured in relation to villus tips. Imaris (version 7.6.3, Bitplane) was used for image analysis and processing.

### Isolation of total RNA

Upon collection, colons were flushed with ice-cold PBS and submerged in RNAlater™ stabilization solution (Invitrogen™) for 24 h in room temperature before they were stored at − 80 °C. For RNA extraction, colons in RNAlater™ were thawed on ice and cut open longitudinally. Colons were divided into three equally sized segments, of which the colonic mucosa of the distal segment was scraped using a microscope glass slide. The mucosal scrapings were transferred to Eppendorf tubes and kept on RNAlater™ at room temperature until RNA isolation. Total RNA was isolated from the colonic scrapings using the NucleoSpin RNA/Protein kit (Macherey–Nagel), following the manufacturer’s instructions. Quantity and integrity of the isolated RNA was assessed by Nanodrop™ 2000c (Thermo Scientific) and Agilent 2100 BioAnalyzer (Agilent Technologies).

### RNA-Seq and data processing

The 19 samples of total RNA subjected to RNA-Sequencing (RNA-Seq) had 260/230 ratios ranging from 1.20 to 2.14, and RIN values ranging from 6.9 to 9.9. Electropherograms and gels produced by Bioanalyzer showed distinct peaks/bands corresponding to 18S and 28S ribosomal RNA (Supplementary Figure S1). Library preparation and sequencing were conducted by the Norwegian Sequencing Centre (NSC). Briefly, libraries were generated using the TruSeq Stranded mRNA Library Prep Kit (Illumina Inc.) according to manufacturer’s manual. Sequencing was performed on an Illumina NovaSeq 6000 SP system using a single end 100 bp run. The yielded number of reads per sample ranged from 39 092 586 to 87 826 915 (Supplementary Table S1).

The quality of the RNA-Seq data was assessed using FastQC^21^ and MultiQC^22^. The reads were adapter trimmed with Trim Galore (v. 0.6.5) and mapped to the mouse reference genome GRCm38.p6 using HISAT2 (v. 2.1.0)^23^. The overall alignment rates were above 96% for all samples (Supplementary Table S1). The BAM files generated by HISAT2 were then imported and visualized in SeqMonk v1.47.1 (available from https://www.bioinformatics.babraham.ac.uk/projects/seqmonk/), specifying a minimal mapping quality of 20. The RNA-Seq quantitation pipeline implemented in SeqMonk was used to quantitate the read counts. Quantitation was conducted at the gene level by counting the merged transcripts over exons with 75-percentile normalization of all libraries. The final quantitated values were presented as log2 transformed reads per million reads (RPM). Functional enrichment analysis was performed with g:Profiler (version e102_eg49_p15_7a9b4d6) against a custom background list of expressed genes (at least one read detected in at least one sample), with Benjamini–Hochberg FDR correction applying significance threshold of 0.05^24^ (Supplementary Tables S3-S4). The DEGs *Gm1574*, *D330028D13Rik*, *Clca5*, *Clca3*, *1810030J14Rik*, *Gm11062* and *Spna1* were presented with their updated names: *Stmnd1*, *Fam221a*, *Clca2*, *Clca1*, *Mptx1*, *Mptx2* and *Spta1*, respectively.

The BAM files were deposited to the Sequence Read Archive and are available under the accession number PRJNA783312.

### Real-time quantitative PCR (RT-qPCR)

Total RNA isolated from colonic scrapings was reverse transcribed into cDNA using the iScript cDNA synthesis kit (Bio-Rad Laboratories Inc.). RT-qPCR was performed with cDNA equivalent to 6 or 12 ng RNA in a 20 µL reaction mix using Ssofast Evagreen Supermix (Bio-Rad Laboratories Inc.) on a Bio-Rad CFX96 Touch Real-Time PCR instrument (Bio-Rad Laboratories Inc.) using following program: Enzyme activation 95 °C (1 min) and 40 cycles of denaturation 96 °C (5 s) and annealing and elongation 60 °C (20 s). Melting curves were included to evaluate primer specificities. Assay primers (exon-exon junction spanning) were designed with the Standard BioTools D3 Assay Design Portal (https://d3.standardbio.com/). Primers were validated using the same reagents and parameters on the Bio-Rad CFX96 Touch Real-Time PCR instrument as above. Primer efficiencies were estimated by LinRegPCR analysis software^25,26^, all demonstrating Ê and R2 within the acceptable range (Ê = 2 ± 0.1 and R2 > 0.98). Primer sequences are listed in Supplementary Table S2.

### Microbial community analyses

Caeca were snap frozen in liquid nitrogen immediately after collection and stored at − 80 °C until DNA extraction. DNA was extracted as previously described^27^, including mechanical lysis by bead-beating. Amplicon libraries were prepared via a two-step PCR amplifying the V3-V4 regions, as described in detail previously^28^. Amplicons were purified with the AMPure XP system (Beckman Coulter) before sequencing. High-throughput amplicon sequencing was performed at the ZIEL Institute for Food & Health, Technical University of Munich, according to previously described procedures^27^. Sequencing was carried out in a paired-end mode (PE300) using a MiSeq system (Illumina Inc.).

The analyzed 16S rRNA gene (V3–V4) amplicon dataset included 416, 73 high-quality and chimera-checked sequences (7956–45 498 per sample), which represented a total of 183 OTUs. Raw reads were processed with the Integrated Microbial Next Generation Sequencing pipeline^29^, based on the UPARSE approach^30^. Briefly, sequences were demultiplexed, trimmed to the first base with a quality score > 3, and assembled. Sequences with < 300 and > 600 nucleotides, as well as assembled sequences with expected error > 3 were excluded from the analysis. Remaining reads were trimmed by 10 nucleotides at forward and reverse end to prevent analysis of regions with distorted base composition. The presence of chimeras was tested with UCHIME^31^. Operational taxonomic units (OTUs) were clustered at 97% sequence similarity (USEARCH 11.0)^32^, and only those with a relative abundance > 0.25% in at least one sample were kept. Non 16S sequences was removed by use of SortMeRNA (v4.2)^33^ with SILVA release 128 (https://www.arb-silva.de/documentation/release-128/) as reference. Sequence alignment and taxonomic classification at 80% confidence level was conducted with SINA 1.6.1^34^ using the taxonomy of SILVA release 128. Phylogenetic tree was generated with Fasttree^35^. Specific OTUs were identified using EzBioCloud^36^.

Raw fastq sequence files were deposited to the Sequence Read Archive and are available under the accession number PRJNA783312.

### Statistical analyses

Differentially expressed genes (DEGs) were identified from raw read counts using DESeq2 corrected for multiple testing^37^ in the R programming language (R version 4.0.2)^38^ implemented in SeqMonk. For the RT-qPCR data, Cq values were normalized to *Gapdh* and the relative expression levels were calculated by the comparative threshold (ΔΔC_T_) method with the Lab group as control group.

Microbial profiles and composition were analyzed in the R programming environment using Rhea^39^ (available from: https://github.com/Lagkouvardos/Rhea). OTU tables were normalized to account for differences in sequence depth by division to their sample size and then multiplication by the size of the smaller sample. Beta-diversity was computed based on generalized UniFrac distances^40^, and the significance of separation between groups was tested by permutational multivariate analysis of variance (PERMANOVA). Alpha-diversity was assessed based on species richness and Shannon effective diversity as explained in detail in Rhea. Only taxa with a prevalence of ≥ 30% (proportion of samples positive for the given taxa) in one given group, and relative abundance ≥ 0.25% were considered for statistical testing. Statistical differences in abundance and prevalence between two groups were determined by Wilcoxon Rank Sum test and Fisher’s Exact test, respectively.

Statistical analyses were performed using the R programming environment or GraphPad Prism 6 (v6.07; GraphPad Software Inc.; San Diego, CA, USA). All applied statistical methods are specified in figure legends. Prior to application of parametric statistics, normality and homogeneity of variance was tested on residuals by Shapiro–Wilk and Levene’s tests, respectively. Heatmap and dotplot was created using the *heatmap.2* and *geom_point* functions from the gplots^41^ and ggplot2^42^ packages in R, respectively. Figures were created using GraphPad Prism 6 (v6.07; GraphPad Software Inc.; San Diego, CA, USA) and Inkscape (v0.92.4; http://www.inkscape.org/).

## Results

### Microbial exposure via feralization did not significantly alter colonic nor ileal mucus layer properties

**Figure 2:**
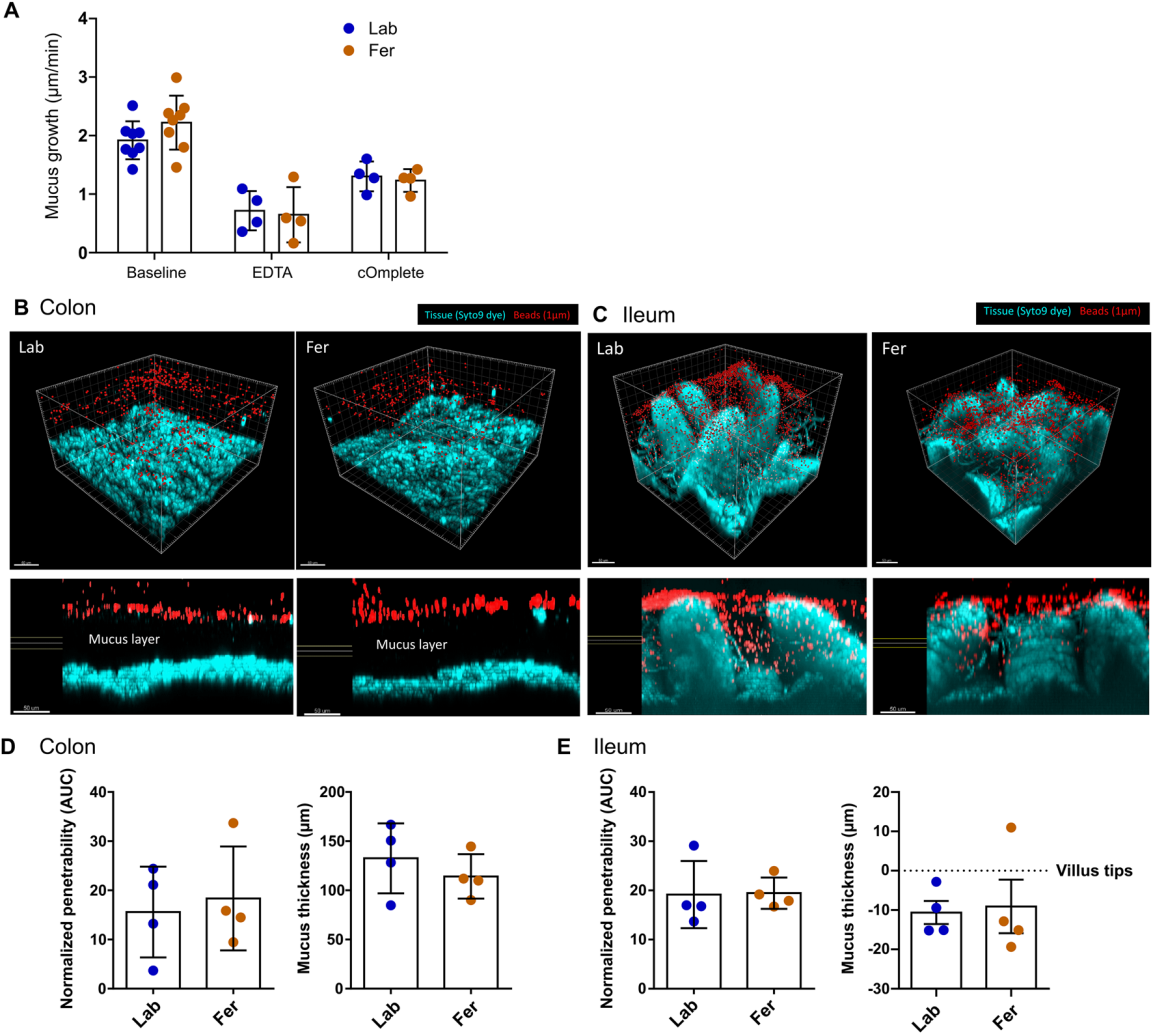
Assessment of intestinal barrier function in male Fer and Lab mice by measurements of mucus layer properties in colon and ileum at week 8. (**A**) Mucus thickness was measured using needle over time in an ex vivo perfusion system, first in the absence (Baseline, n = 8/group) then in presence of protease inhibitors (EDTA; metalloprotease inhibitor (n = 4/group), cOmplete EDTA-free cocktail; serine and cysteine protease inhibitor (n = 4/group)). Mucus growth was expressed as µm mucus growth/minute. Significance was determined by two-way ANOVA followed by Tukey’s multiple comparisons test for significant main effect. **p < 0.01, ***p < 0.001 difference from Baseline. (**B, C**) Thickness and penetrability of mucus layers in colon and ileum of male Fer (n = 4) and Lab (n = 4) mice. The mucus barrier properties were measured by assessing penetration of bacteria-sized beads via confocal microscopy. Pictures show 3D overviews of confocal z-stacks (top) and cross-section view (bottom) of colon (**C**), and ileum (**D**), with corresponding bar plots of results (**D, E**). Fer, feralized; Lab, laboratory.

After 8 weeks of feralization, mice were subjected to assessment of intestinal mucus layer properties. We found no difference in baseline growth rate between Fer and Lab mice, and a significant decrease in growth in both groups in response to inhibitor treatment (Fig. 2A). Barrier function expressed as normalized penetrability (bead distribution within mucus) and mucus thickness (average tissue-bead distance) were similar between the two groups in both colon and ileum (Fig. 2B–E), suggesting that feralization by housing in a farmyard-type habitat had no major influence on intestinal mucus layer properties. In ileum, segmented filamentous bacteria (SFB) could be seen as filaments between the villi (Fig. 2C). These were present in both groups, although visual evaluation was suggestive of them being more abundant in the Fer group.

### Transcriptome profiling revealed barrier-related genes in colonic tissue were differentially expressed according to microbial exposure

**Figure 3:**
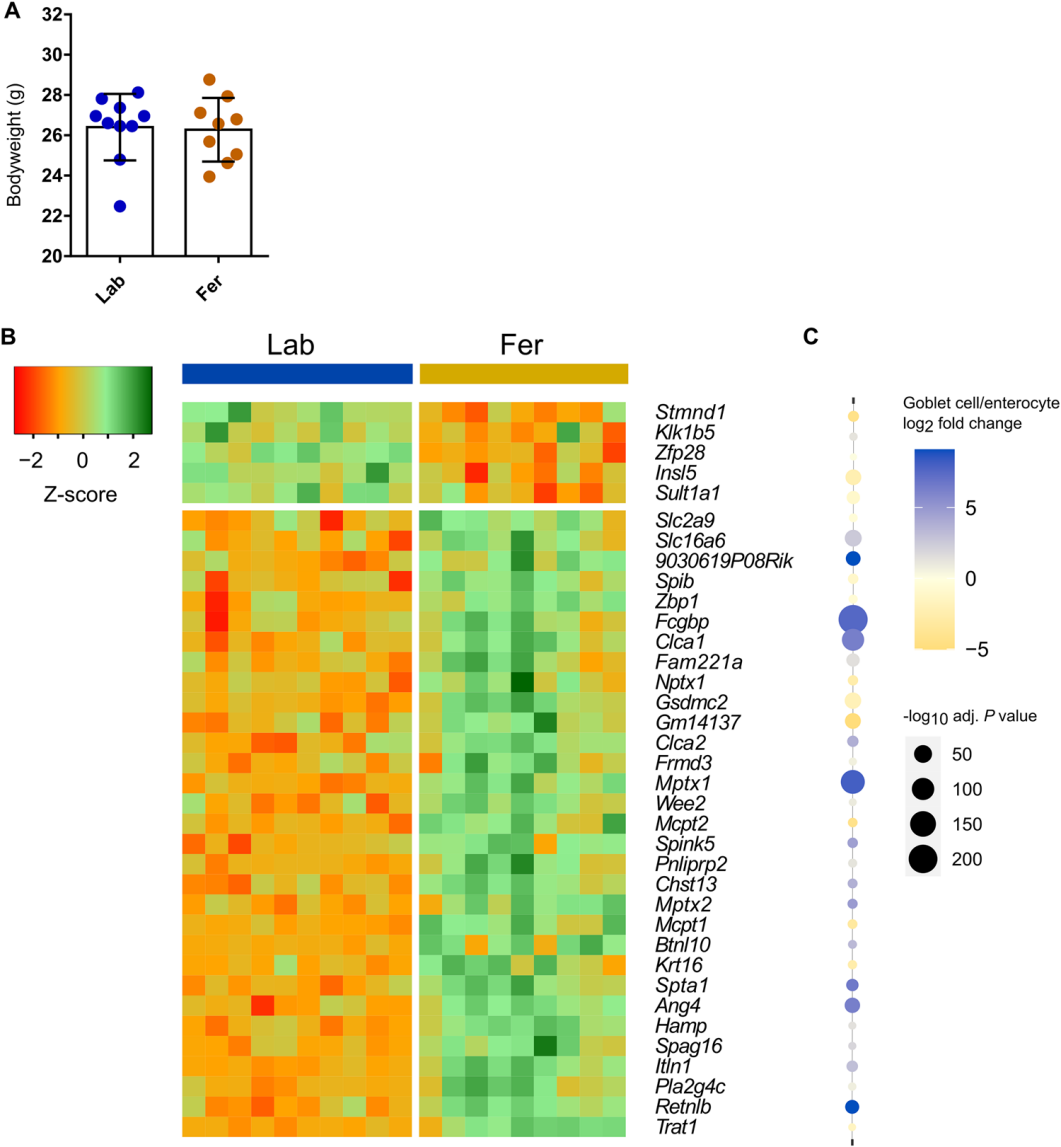
Bodyweight registration **(A)** and differentially expressed genes in the colonic mucosa of feralized and lab mice (**B**) terminated at week 12. Heatmap of DEGs showing significant (*p* < 0.05, FDR adjusted) differences in gene expression between the two groups, displayed using log_2_ transformed normalized counts from DESeq2, scaled to Z-score for each gene. Fer, feralized; Lab, laboratory. See also Supplementary Figure S2. (**C**) Dot plot showing relative expression levels of the DEGs in B in goblet cells versus enterocytes as reported in^46^. Colors represent log_2_ fold change values while size of dots represent significance levels (− log_10_ adjusted *P* values).

Mice feralized for 12 weeks exhibited no differences in bodyweight compared to Lab mice (Fig. 3A). Mucosal scrapings from colon tissues collected from Fer and Lab were subjected to RNA isolation and subsequent RNA-Seq. The RNA-Seq identified 31 significantly upregulated and 5 significantly downregulated genes in Fer mice compared to Lab mice (Fig. 3B; Supplementary figure S2).

Among the upregulated genes in the Fer mice (Fig. 3B) we detected genes encoding known goblet cell products involved in mucus layer organization and generation. *Fcgbp* and *Clca1*, both upregulated in Fer mice, encode Fcgbp (IgGFc-binding protein) and Clca1 (calcium-activated chloride channel regulator 1) that have been shown to constitute the major proteins in colon and ileum mucus together with the Muc2 mucin^43–45^. Other genes expressed in epithelial cells that were found more expressed in Fer mice compared to Lab mice encode proteins holding antimicrobial and inflammatory functions; *Itln1*, *Ang4* and *Retnlb* encoding the antimicrobial proteins Intelectin 1, Angiogenin 4 and Resistin-like molecule β, and *Zbp1* and *Gsdmc2* encoding the inflammation-related proteins Z-DNA-binding protein 1 and Gasdermin C. These seven genes related to intestinal barrier function (*Clca1*, *Fcgbp*), antimicrobial functions (*Itln1*, *Ang4*, *Retnlb*) and inflammation (*Zbp1*, *Gsdmc2*) identified as DEGs from RNA-Seq were checked with traditional RT-qPCR, largely confirming the RNA-Seq results (Supplementary figure S3). Additionally, we included *Alpi*, a gene encoding intestinal alkaline phosphatase and that was not identified as a DEG from RNA-Seq, as a negative control in the RT-qPCR analyses. We confirmed no differential expression of *Alpi* between the two experimental groups. By comparison of the DEGs with previously published gene expression data from purified goblet cells^46^, we found that most genes upregulated in our Fer mice were goblet cell specific (Fig. 3C).

To better explain the biological function of the differentially expressed genes (DEGs), we conducted a functional enrichment analysis. Functional enrichment analysis of the 31 genes upregulated in Fer mice identified by DESeq2 were associated with GO terms such as: response to biotic stimulus (adj. *P* = 0.007), defense response to bacterium (adj. *P* = 0.017), biological process involved in interspecies interaction between organisms (adj. *P* = 0.014) and response to other organism (adj. *P* = 0.007) (Supplementary Figure S4; Supplementary Table S3). A similar GO analysis of the five genes upregulated in Lab mice associated with terms such as chemical carcinogenesis (adj. *P* = 0.039), estrogen metabolism (adj. *P* = 0.011) and retinol metabolism (adj. *P* = 0.017) (Supplementary Figure S5; Supplementary Table S4).

### Caecal microbiota profile of feralized mice differed significantly from that of laboratory mice

**Figure 4:**
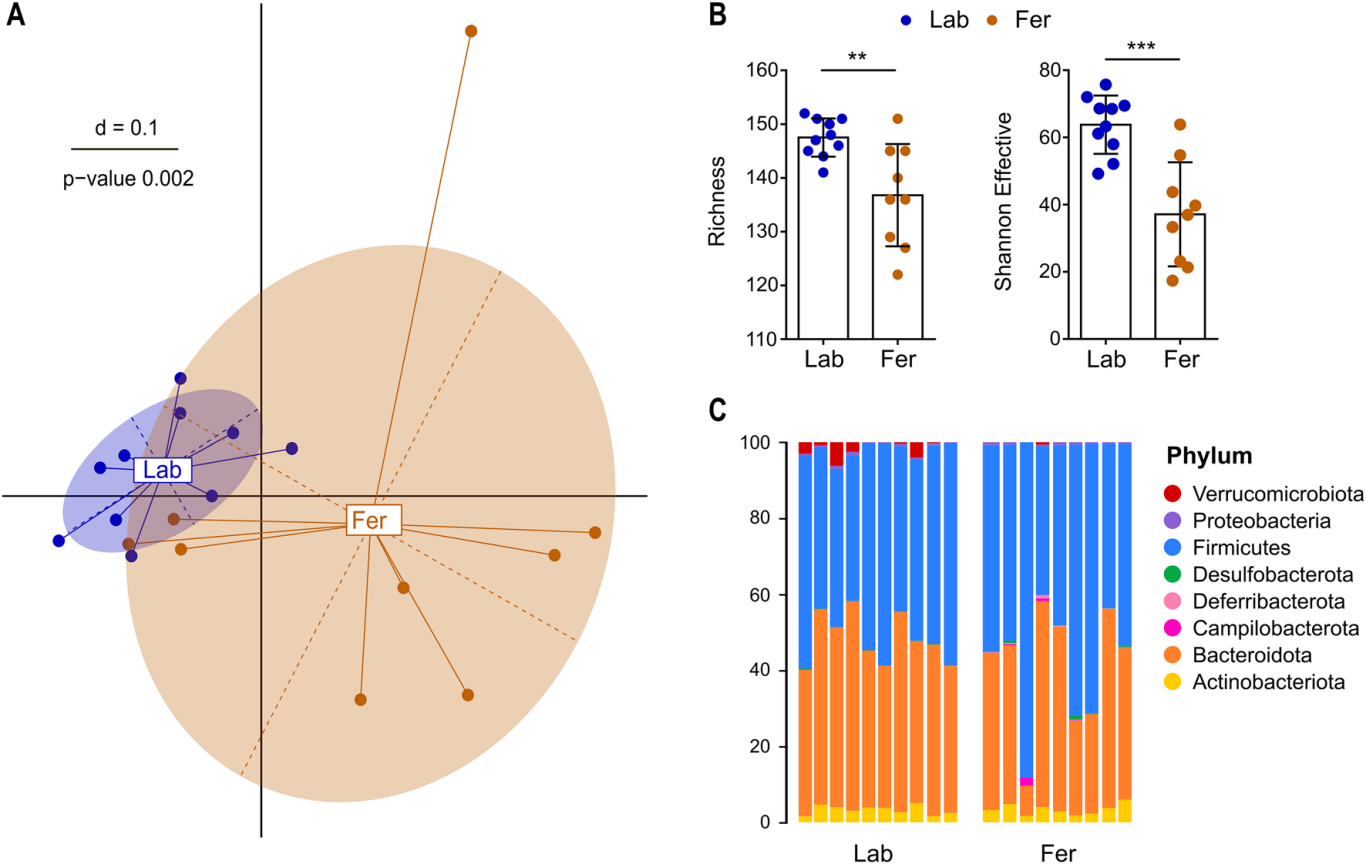
Caecal microbiota profiles and composition of Fer and Lab mice. (**A)** Multi-dimensional scaling (MDS) plot of fecal microbiota profiles (generalized UniFrac distances) for Fer and Lab mice terminated at week 12. Significance of separation was determined by PERMANOVA. (**B**) Observed number of OTUs (Richness) and Shannon Effective counts for all groups. Plots show group mean (bars), SD (error bars) and individual mice (dots). Significant differences between the groups were determined using unpaired t-test. **P < 0.01; ***P < 0.001. (**C**) Taxonomic binning at the rank of phylum, presented as relative abundance for each individual. Fer, feralized, n = 9; Lab, laboratory, n = 10.

Laboratory tests for common mouse pathogens were negative in fecal samples from mice representative for the Fer as well as the Lab groups as reported previously^4^. Likewise, standard examination (McMasters and immunofluorescent antibody testing for Cryptosporidium and Giardia) of mouse feces for parasites were negative^4^. Microbial community analysis was conducted on caecal samples from the mice terminated at week 12 and *Beta*-diversity analysis identified significant clustering of the two groups, albeit the inter-individual variance in the Fer group was greater than the Lab group (Fig. 4A). The richness and effective Shannon counts were significantly lower in the Fer group than the Lab group (*P* = 0.004 and *P* < 0.001, respectively) (Fig. 4B), indicating a lower diversity of the Fer microbiota.

No significant differences in relative abundances were detected at the phylum level (Fig. 4C). However, the phylum Campilobacterota was only present above cutoff for statistical analyses (≥ 0.25%) in the Fer mice (3/9). This phylum was represented by a single OTU showing the closest sequence similarity to *Helicobacter equorum* (99.5%). Moreover, Verrucomicrobiota was present above cutoff in 6/10 Lab mice but only 1/9 Fer mice. This phylum was represented by a single OTU showing the closest sequence similarity to *Akkermansia muciniphila* (99.5%). At the genus level, Fer mice had significantly lower relative abundance of *Alistipes*, and significantly lower prevalence *Dubosiella* (9/10 Lab, 3/9 Fer) and *Faecalibacterium* UBA1819 (10/10 Lab, 3/9 Fer).

## Discussion

The objective of this study was to examine the impact of microbial exposure through feralization of research mice on intestinal barrier function. We previously reported protective effects of feralization on colorectal cancer development^4^ and hypothesized this could be explained, at least in part, by changes in the local barrier function prior to cancer induction. We have previously found that free-living feral mice have thick, impenetrable mucus layers^17^ and hypothesized that the higher environmental microbial load resulting from feralization would strengthen the mucus layer similar to wild mice. However, our current study found no changes in mucus quality resulting from 8 weeks of microbial exposure in our model. Several notable differences remain between captured wild mice and feralized mice that could contribute to explain the incompatible findings, such as unknown age, infectious history and ubiquitous presence of intestinal parasites, the latter which was not detected in our feralized mice^4^.

Although RNA-Seq of colonic mucosa after 12 weeks of microbial exposure in our model revealed relatively few DEGs between the two groups, the functional enrichment analysis pointed towards the direction of defense mechanisms for the genes significantly upregulated in Fer mice. A closer look at the genes upregulated in Fer encode two of the three major colonic mucus proteins, Fcgbp and Clca1^43,44^. The functional role of Fcgbp and Clca1 are incompletely understood. Interestingly, these proteins are not found in a normal respiratory tract but appear and become as high levels as in colon mucus upon the formation of an attached mucus layer^47^. Fcgbp (IgGFc-binding protein) does not, despite its name, bind to immunoglobulins. Rather, it forms large polymers that are likely involved in mucus attachment^46,48^, and has been suggested to hold an endogenous protective role in wound healing upon colitis induction in mice^49^. Clca1 (calcium-activated chloride channel regulator 1, previously Clca3 in mice) acts as a metalloprotease in the mucus and is likely involved in structural rearrangements of the mucus layer^19,50^. We did not observe any differences in the mucus penetrability or growth of mucus, but this does not exclude that there are substantial alterations in the Fer mucus. Such conclusions are also supported by the observation of less colon cancer development in the Fer mice^4^ as protection of the epithelium and suppression of inflammation are known to lower the risk for cancer^51^. The mice subjected to RNA-Seq were feralized for approximately four weeks longer than the mice subjected to mucus measurements, which may in part explain the discrepancies. To elaborate on the in vivo performance of the feralized mice intestinal barrier future studies should consider assessing permeability of the epithelium (e.g., administration of FITC-Dextran^52^), record the distance between bacteria and the epithelium in Methanol-Carnoy fixed tissue to assess the functional mucus penetrance of commensal bacteria^53^, and finally perform experiments that test the functional barrier property against pathogen infections (e.g., C. rodentium) or chemically induced mucus degradation by e.g., dextran sulfate sodium.

Other genes expressed in epithelial cells that were found upregulated in Fer mice encode proteins with antimicrobial functions, such as Intelectin1, Angiogenin 4 and Resistin-like molecule β. Intelectin 1 is a protein that binds to bacterial glycans and aggregates bacteria^54,55^. This protein is likely acting as another mucus protein, Zg16, that also aggregates bacteria and could potentially move bacteria in the mucus layer further away from the epithelium^56^. Resistin-like molecule β contributes to preventing bacterial access to intestinal epithelium as well as being involved in host defense against parasites^57^. Mice depleted of their microbiota exhibit decreased expression of *Ang4* and *Retnlb* in colonic epithelial cells^58^, supporting our findings of increased expression in the microbially experienced Fer mice.

Genes related to inflammation and immunosurveillance were also upregulated following feralization, such as *Gsdmc2* encoding Gasdermin C, which is a known effector protein for pyroptosis. Pyroptosis may play a role in antitumor immunity by facilitating the killing of tumor cells^59,60^. Gasdermin C has mostly been studied in the small intestine. In a recent article, *Gsdmc2* was identified as a target gene in small intestinal epithelial cells for the type 2 cytokines IL-4 and IL-13, and the authors suggested that the Gsdmc family of proteins could be important effectors for type 2 responses in the gut^61^. *Zbp1* is another gene that was upregulated in Fer mice. This gene encodes Z-DNA-binding protein 1, identified as an innate sensor of viral infections that is induced by IFN with effects including regulation of cell death and inflammation^62^. Upregulation of the Zbp1 gene could not be confirmed by RT-qPCR, possibly due to binding of primers to alternative transcript variant(s) shorter in size that display preferential amplification in RT-qPCR. *Alpi*, a gene encoding intestinal alkaline phosphatase, was not differentially expressed in RNA-Seq and the RT-qPCR targeting the *Alpi* transcript confirmed this, supporting the data from the former.

Microbiota profiling of caeca was conducted to characterize if differences in the expression of intestinal barrier-related genes according to microbial exposure is reflected in distinct compositions in the feralized mouse gut. In our previous studies, feralization was accompanied by large shifts in fecal microbiota profiles, both when the mice were feralized in the presence^6^ and absence^4^ of wild-caught feral mice. In the current study, analysis of caecal microbiota profiles also showed significant clustering according to microbial exposure. However, minimal differences in composition were found between the Fer and Lab mice. An OTU with closest sequence similarity to *Helicobacter equorum* was only detected in our Fer mice, a bacterial species found in horses^63^ and thus may have originated from horse fecal matter in the farmyard-type environment. *Helicobacter spp.* are frequently detected in wild mice^13,64^, suggesting they are natural components of a wild mouse microbiota. *Helicobacter spp.* have also been consistently detected in our previous feralization studies^4,6^, albeit direct comparisons between the current and previous feralization studies should be made with caution, as they differ both in the tissues sampled and gender of the mice—parameters that have been described to convey changes to the microbiota composition and functions^65,66^. The relationship between gut microbiota components and intestinal barrier is highly complex, and while some bacterial species and their metabolites have been associated with enhanced and diminished intestinal barrier function^67^, the interplay of entire microbiome community structures and intestinal barrier structures remains unresolved. The effects of feralization on intestinal barrier is likely mediated via the microbiota and its metabolites, yet to better understand this interplay, future studies should aim to standardize the analysis by collecting samples for metagenomic, metabolomic and transcriptomic analyses from a defined intestinal region, to generate more comprehensive data.

An interesting note requiring further investigation was signs of a higher presence of SFB observed in the ileum of Fer mice by subjective confocal microscopical assessment. SFB are known as one of few non-pathogenic bacteria that penetrate the mucus layer. SFB adhere to intestinal epithelial cells and have been shown to induce non-inflammatory Th17 responses^68^ producing IL-17A and IL-22 that in turn promote expression of antimicrobial peptides and regulation of tight junction proteins^69,70^. Thus, microbial profiling of intimate mucosa to empirically detect potential differences in presence of SFB should be considered in future studies.

The RNA-Seq was conducted on mucosal scrapings from one section of the colon, representing overall colonic mucosal gene expression levels from this intestinal section. Most of the DEGs we identified have been previously reported as goblet cells specific^46^, although we did not here determine gene expression in single cell populations. Future studies should aim to include careful isolation of the epithelium and lamina propria followed by targeted approaches to obtain transcriptomic data specific for various cell types using single-cell transcriptome sequencing. Additionally, an important note is that mRNA does not always correspond to protein levels^71^, thus the functional consequences of the differentially expressed genes remains to be investigated by analyses of protein content. Finally, our transcriptome analyses covered only a limited part of the colon, and thus we recommend that other parts of the intestine are investigated in future studies.

In conclusion, we here described how microbial exposure through housing a group of juvenile male C57BL/6JRj mice in a farmyard-type habitat influenced the mucus layer function and intestinal barrier gene expression. Although prominent effects on growth rate, thickness and penetrability were not found, the increased expression of barrier-related genes is suggestive of an enforced intestinal barrier in the feralized mice. Further studies will be required to obtain more in-depth information on the differences between feralized and laboratory mice, to investigate effects on female mice and other mouse strains, and to determine the influence of microbial stimuli on the intestinal barrier and downstream effects on intestinal pathologies such as CRC development.

### Supplementary Information

Supplementary Information 1.

Supplementary Information 2.

Supplementary Information 3.

## Data Availability

All data generated or analyzed during the current study are available from the corresponding author on reasonable request. Sequencing data are available from the Sequence Read Archive under the accession number PRJNA783312.
