# Supplementary material for: Microbial experience through housing in a farmyard-type environment alters intestinal barrier properties in mouse colons
Source: Sci Rep. 2023 Aug 22;13:13701. doi: 10.1038/s41598-023-40640-5 (PMC10444815; doi:10.1038/s41598-023-40640-5)
Supplement: Supplementary file 1 — Supplementary Information 1. [file 41598_2023_40640_MOESM1_ESM.docx]

SUPPLEMENTARY MATERIAL

***Microbial experience through housing in a farmyard-type environment alters intestinal barrier properties in mouse colons***

Henriette Arnesen^1,2^, Turhan Markussen^1^, George Birchenough^3^, Signe Birkeland^2^, Elisabeth Nyström^3^, Gunnar C. Hansson^3^, Harald Carlsen^2^ and Preben Boysen^1*^

^1^Faculty of Veterinary Medicine, Norwegian University of Life Sciences (NMBU), Ås, Norway

^2^Faculty of Chemistry, Biotechnology and Food Science, Norwegian University of Life Sciences (NMBU), Ås, Norway

^3^Mucin Biology Group, Department of Medical Biochemistry & Cell Biology, University of Gothenburg, Sweden

# FIGURES


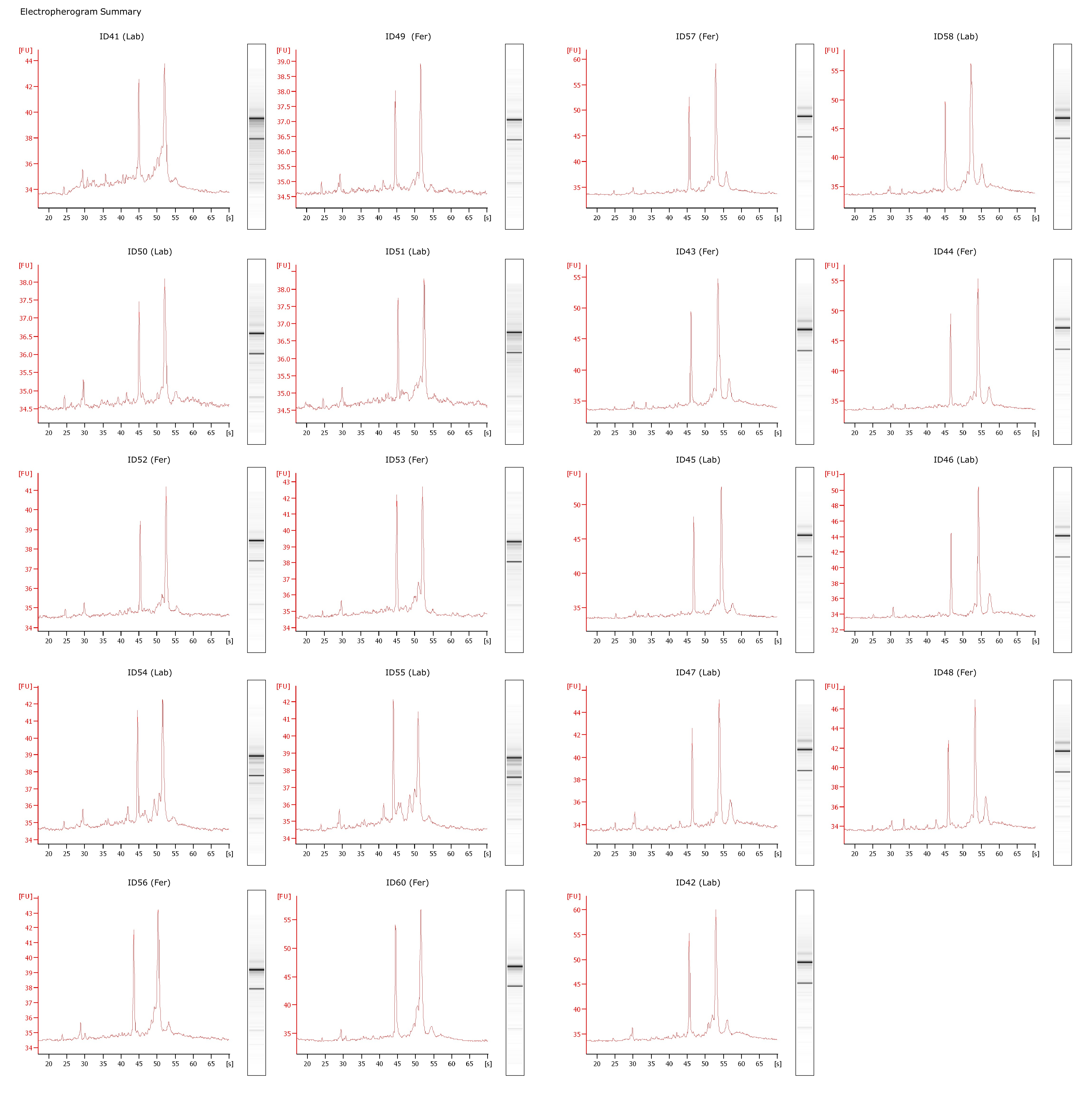


**Figure S1**. Electropherogram summary for samples subjected to RNA sequencing and RT-qPCR.


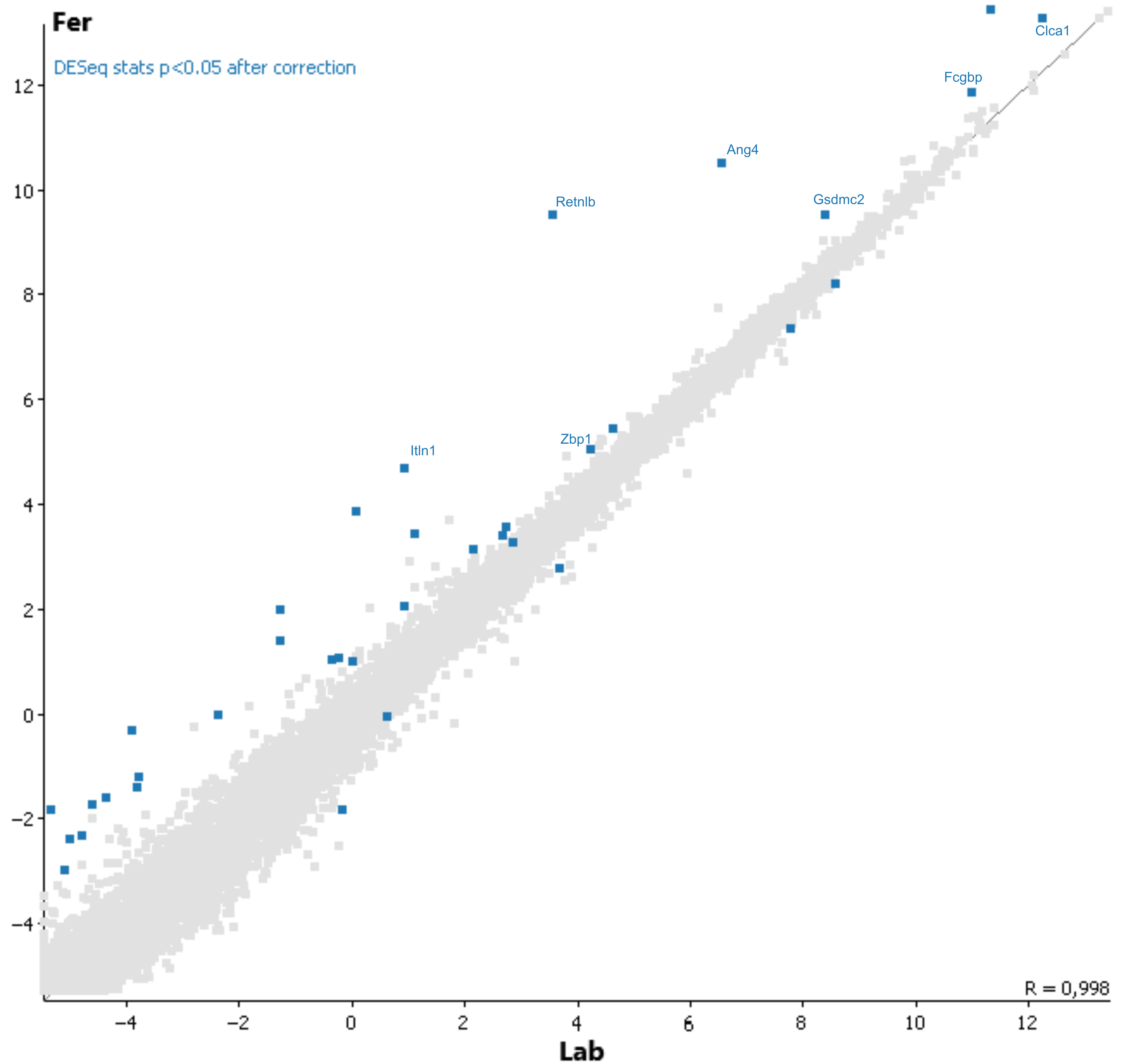


**Figure S2**. Scatterplot of relative expression levels as log_2_ scale RPKM (reads per kilobase transcript per million mapped reads) in Fer and Lab mice. Genes significantly up/down-regulated between the two groups are highlighted in blue (DESeq2 test with FDR cutoff of 0.05).





**Figure S3**. Relative expression of genes in colonic mucosal scrapings determined by RT-qPCR. Data show 2^-ΔΔCq^ values presented as mean (bar) with SD (whiskers) and dots representing individual mice. Significant differences between the groups were determined using unpaired t-tests. *P<0.05 **P<0.01; ***P<0.001. n=6-9/group.


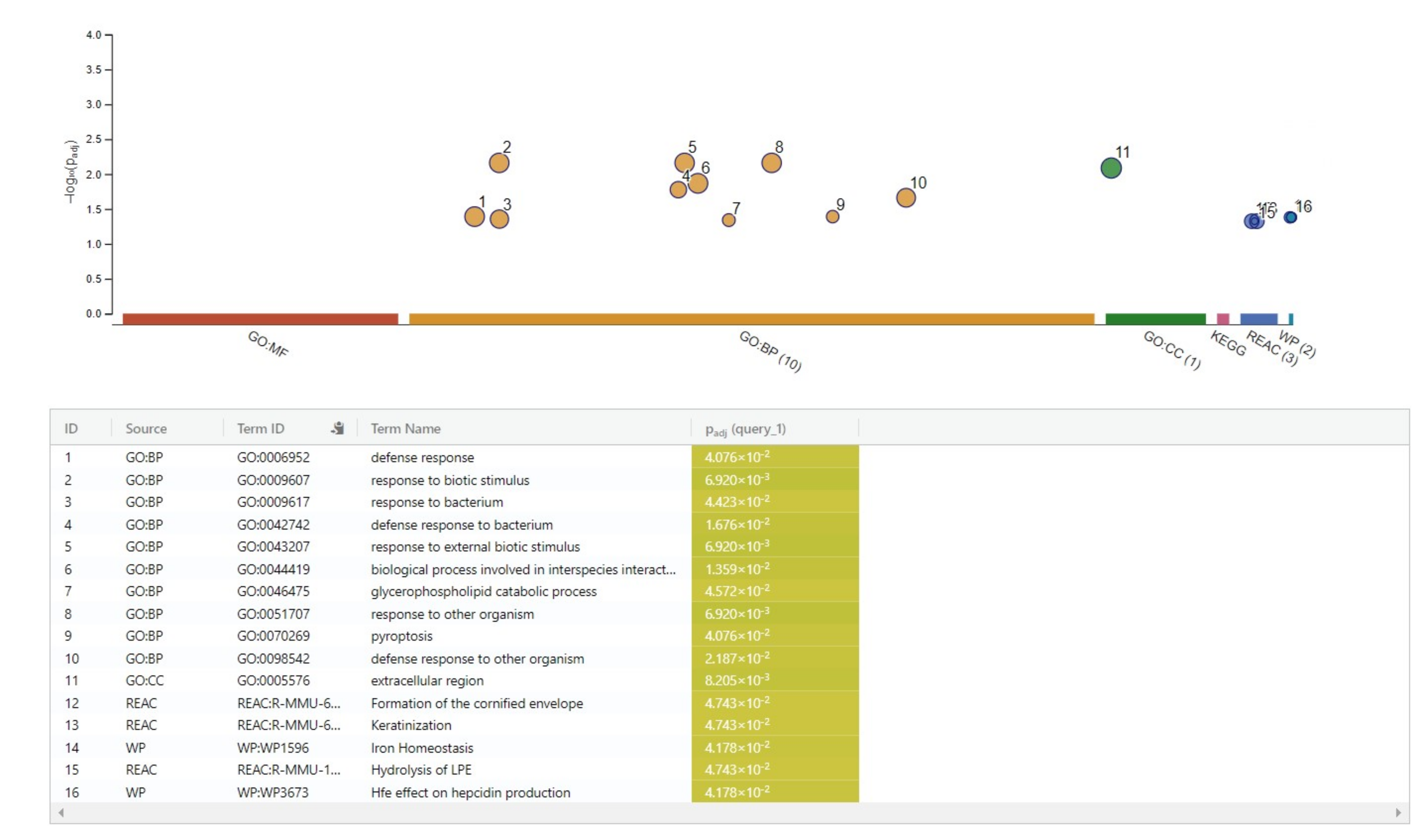


**Figure S4.** Manhattan plot of the significantly enriched gene function annotations from the indicated databases (cutoff *P*<0.05) for the genes significantly upregulated in Fer against a custom list of expressed genes. See also Supplementary Table S3. GO: Gene Ontology, MF: Molecular Function,BP: Biological Process, CC: Cellular Component, KEGG: KEGG Pathway, REAC: Reactome, WP: WikiPathways.


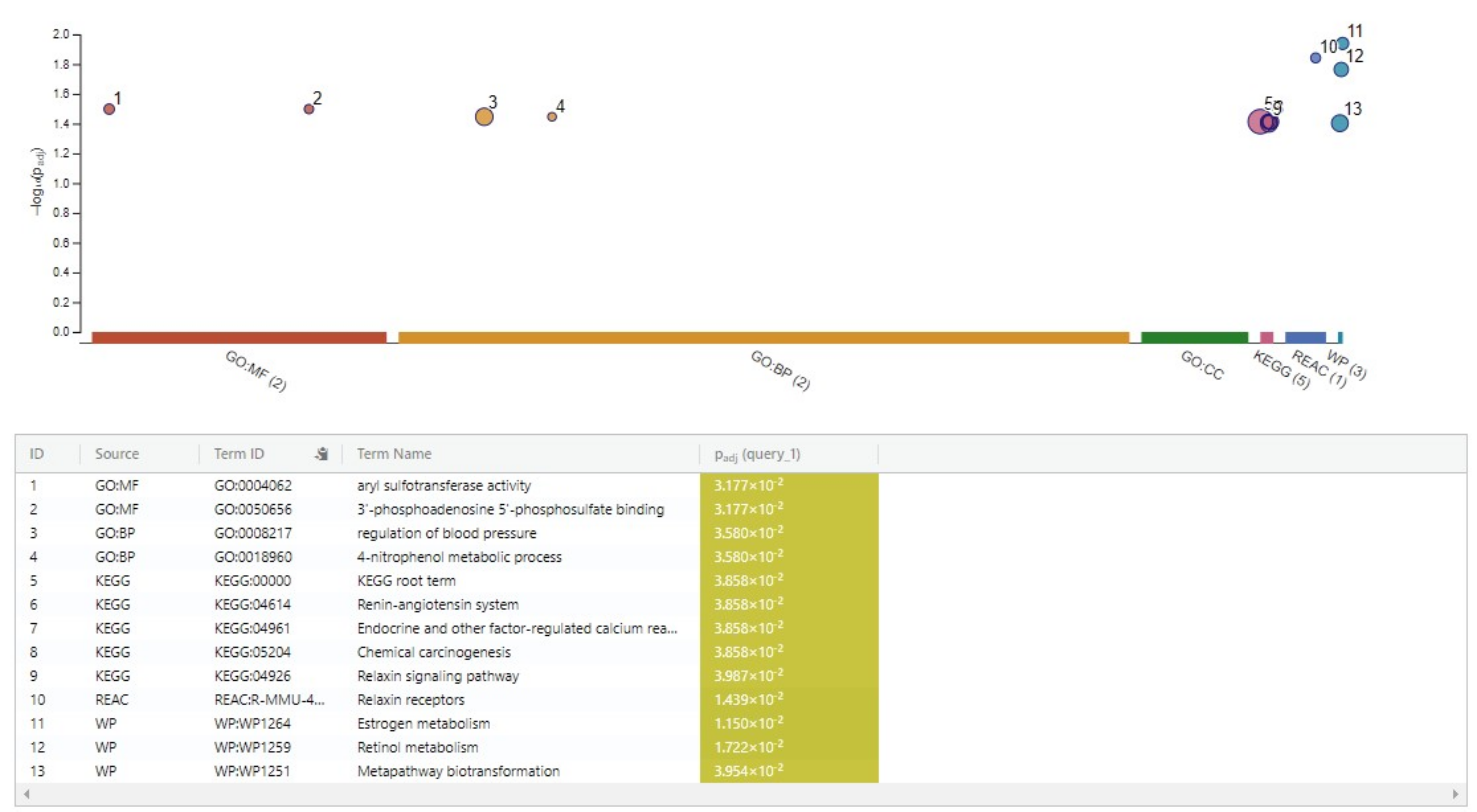


**Figure S5.** Manhattan plot of the significantly enriched gene function annotations from the indicated databases (cutoff *P*<0.05) for the genes significantly upregulated in Lab against a custom list of expressed genes. See also Supplementary Table S4. GO: Gene Ontology, MF: Molecular Function,BP: Biological Process, CC: Cellular Component, KEGG: KEGG Pathway, REAC: Reactome, WP: WikiPathways.

# TABLES

**Supplementary Table S1**. Total number of reads and overall results from alignment to the mouse reference genome GRCm38.p6 by use of HISAT2.

| **Sample** | **Group** | **Total # reads** | **Unpaired reads** | **Aligned 0 times** | **Aligned exactly 1 time** | **Aligned >1 times** | **Overall alignment rate (%)** |
| --- | --- | --- | --- | --- | --- | --- | --- |
| 1 | Lab | 49330089 | 49330089 | 1420377 | 38860131 | 9049581 | 97.12 |
| 2 | Lab | 46394358 | 46394358 | 1192355 | 37458804 | 7743199 | 97.43 |
| 3 | Fer | 45316186 | 45316186 | 869665 | 36788546 | 7657975 | 98.08 |
| 4 | Fer | 57191455 | 57191455 | 1228596 | 46636326 | 9326533 | 97.85 |
| 5 | Lab | 58635434 | 58635434 | 1736077 | 46486755 | 10412602 | 97.04 |
| 6 | Lab | 63742588 | 63742588 | 1519754 | 51942168 | 10280666 | 97.62 |
| 7 | Lab | 45500650 | 45500650 | 1087493 | 37093515 | 7319642 | 97.61 |
| 8 | Fer | 52698880 | 52698880 | 1246776 | 41931132 | 9520972 | 97.63 |
| 9 | Fer | 43487149 | 43487149 | 1095474 | 33847968 | 8543707 | 97.48 |
| 10 | Lab | 54730333 | 54730333 | 1607576 | 41514658 | 11608099 | 97.06 |
| 11 | Lab | 84939759 | 84939759 | 1858172 | 68071185 | 15010402 | 97.81 |
| 12 | Fer | 39092586 | 39092586 | 965914 | 32073569 | 6053103 | 97.53 |
| 13 | Fer | 57072934 | 57072934 | 1755611 | 45311472 | 10005851 | 96.92 |
| 14 | Lab | 48651763 | 48651763 | 1599099 | 38617720 | 8434944 | 96.71 |
| 15 | Lab | 87826915 | 87826915 | 2244022 | 69667739 | 15915154 | 97.44 |
| 16 | Fer | 55516274 | 55516274 | 1789777 | 43309845 | 10416652 | 96.78 |
| 17 | Fer | 45773292 | 45773292 | 1141916 | 36277936 | 8353440 | 97.51 |
| 18 | Lab | 48149835 | 48149835 | 1155817 | 38909424 | 8084594 | 97.60 |
| 20 | Fer | 45764994 | 45764994 | 1102933 | 37093133 | 7568928 | 97.59 |

**Supplementary Table S2. RT-qPCR primers (mouse)**

| **Primer name** | **Full gene name** | **RefSeq ID** | **5’ – 3’ sequence** |
| --- | --- | --- | --- |
| Gapdh-F | Glyceraldehyde-3-phosphate dehydrogenase | NM_008084.3 | CAAGGTCATCCCAGAGCTGAA |
| Gapdh-R | Glyceraldehyde-3-phosphate dehydrogenase | NM_008084.3 | CAGATCCACGACGGACACA |
| Itln1-F | Intelectin 1 | NM_010584.3 | TCTTTTCCTCTCTGCCCAGAA |
| Itln1-R | Intelectin 1 | NM_010584.3 | GTGCGCAGGAAATAGAGACC |
| Clca1-F | Chloride channel accessory 1 | NM_017474.2 | ACAACCACTAAGGTGGCCTA |
| Clca1-R | Chloride channel accessory 1 | NM_017474.2 | GAGCTCGCTTGAATGCTGTA |
| Fcgbp-F | Fc fragment of IgG binding protein | NM_001122603.1 | ATCGAGCAATGTGGCTGCTA |
| Fcgbp-R | Fc fragment of IgG binding protein | NM_001122603.1 | CAATGCTGCTGGCAGTTTTCA |
| Gsdmc-F | Gasdermin C | NM_031378.3 | AGGTTCAGAGTAAGAGCATCCC |
| Gsdmc-R | Casdermin C | NM_031378.3 | ATGTGGGCAACTGATCCAAC |
| Zbp1-F | Z-DNA binding protein 1 | NM_021394.2 | TGGCAGAAGCTCCTGTTGAC |
| Zbp1-R | Z-DNA binding protein 1 | NM_021394.2 | CCAGCTGGCCAATCTTCACA |
| Ang4-F | Angiogenin, ribonuclease A family, member 4 | NM_177544.4 | CTCCAGGAGCACACAGCTA |
| Ang4-R | Angiogenin, ribonuclease A family, member 4 | NM_177544.4 | CAGCACGAAGACCAACAACA |
| Retnlb-F | Resistin like molecule beta | NM_023881.4 | CCTAAGACGATCTCCTGCACTA |
| Retnlb-R | Resistin like molecule beta | NM_023881.4 | AGCACATCCAGTGACAACCA |
| Alpi-F | Alkaline phosphatase, intestinal | NM_001081082.2 | TCGCCACTCAACTCATCTCC |
| Alpi-R | Alkaline phosphatase, intestinal | NM_001081082.2 | AGTCCCCTTGGGAAACATGAA |

**Supplementary Table S3**. Functional enrichment analysis of genes significantly upregulated in the Fer group (xlsx).

**Supplementary Table S4**. Functional enrichment analysis of genes significantly upregulated in the Lab group (xlsx).
